# Group identity modulates bidding behavior in repeated lottery contest: neural signatures from event-related potentials and electroencephalography oscillations

**DOI:** 10.3389/fnins.2023.1184601

**Published:** 2023-06-22

**Authors:** Su Hao, Pan Jiali, Zhang Xiaomin, Wang Xiaoqin, Lu Lina, Qing Xin, Liu Qin

**Affiliations:** ^1^School of Economics and Management, Southwest Petroleum University, Chengdu, China; ^2^Key Laboratory of Energy Security and Low-carbon Development, Chengdu, China

**Keywords:** group identity, contest, overbidding, event-related potentials, event-related oscillations

## Abstract

A contest usually involves expenditures, termed “overbidding,” exceeding the theoretical Nash equilibrium. A considerable number of studies have shown that group identity can affect decision-making and competitive behavior, thus providing a new perspective on alleviating the overbidding problem. How group identity influences brain activity when competitors bid in different groups is not yet clear, however. In this study, we implemented group identity manipulation into the lottery contest game and we recorded behavioral and electroencephalography (EEG) data at the same time. Two experimental treatments were conducted to study the effect of group identity on bidding behavior. The event-related potentials (ERP) and event-related oscillations (ERO) techniques were utilized to explore brain activity differences caused by participants’ different bidding behaviors under in-group and out-group conditions. Behavioral results showed that individual expenditure was significantly lower when bidding with in-group opponents than with out-group opponents. Analyses of EEG results revealed that compared to in-group conditions, greater N2 amplitudes and theta power were found under out-group conditions. To extend previous studies, we performed supplementary analysis to explore whether enhancement of group identity had effects on conflict alleviation. Behavioral results indicated that individual expenditure was significantly lower after enhancing group identity when bidding with in-group, and EEG results showed more negative N2 amplitudes, smaller P3 amplitudes and larger theta power after enhancing group identity. Collectively, these findings indicate that group identity modulated bidding behavior, and they provide insight into a mechanism to de-escalate group conflict by enhancing group identity.

## 1. Introduction

Rent-seeking, that is, manipulating social environments and economic conditions to increase profit without creating new wealth, is an omnipresent social phenomenon in which several agents often compete to obtain a scarce resource—with each competitor exerting costly efforts to increase the probability of doing so. However, these expenditures are sunk and irrecoverable, so they have little social value to both winner and loser (Chakravarty et al., 2016). Because observational field data on rent-seeking behavior are rarely available (Fonseca, 2009; Rockenbach and Waligora, 2016), researchers have turned to numerous experiments and methods to investigate various forms of contests [see a review by Dechenaux et al. (2015)]. According to classical game theory analyses, rational and self-interested participants should make decisions to reach the best outcomes, called Nash equilibrium, from which no participant can increase their payoffs by changing their strategies unilaterally. While decades of experimental studies on contests have demonstrated the robust phenomenon that contestants incurred expenditures exceeding the original theoretical Nash equilibrium level; this is often referred to as “overbidding” (Chowdhury et al., 2014). Several types of contest models have been developed (Cason et al., 2018), for example, proportional-prize contests in which a fixed prize is shared in proportion to performance, winner-take-all contests won by the best performer, and winner-take-all lottery contests in which an exogenously fixed prize is allocated probabilistically in proportion to observable efforts. Proportional-prize contests are less risky and have less variance in payoffs across participants. The Nash equilibrium and observed efforts are consistently the highest in the simple deterministic winner-take-all contest. Lottery contests and proportional-prize contests have the same level Nash equilibrium, but winner-take-all lottery contests induce participants to make greater efforts but receive lower, more unequal payoffs. A large body of reliable empirical evidence shows that people’s views and behaviors are strongly influenced by the membership of groups they belong to and identify with, and they tend to develop attachment feelings and in-group favoritism toward these groups (Brewer, 1979). Group membership (or group identity), quite intuitively, seems to provide an interesting method for potentially alleviating or resolving the overbidding problem.

Sen (2007) considered “identity” an attribute that provides a “strong and exclusive sense of belonging” to a group. In research, group identity is created or induced according to certain experimental designs that break through subjects’ inherent social identity (e.g., nationality, country, race, gender). Previous economic studies have revealed that identity induced in a laboratory can affect behavior; moreover, extensive studies have found that social or group identity influences individuals’ decision-making (Le Coq et al., 2015) and market behaviors (Li et al., 2011). Tajfel et al. (1971) developed an experimental paradigm, termed the “minimal group paradigm,” to prove that, among groups, social categorization is sufficient *per se* to cause discrimination. Many experiments have replicated the “minimum group case” that randomly and arbitrarily assigns individuals to groups, generating identification with members of their own group and often hostility toward members of other groups. Previous study designs have classified groups by recognizable figurative symbols, for instance, wearing different badges or different colored T-shirts (Morita, and Serv’atka, M., 2013; Chowdhury et al., 2016). Some designs were based on subjects’ preference for pictures or words (Chen and Li, 2009; Masella et al., 2014), even some randomly and arbitrarily assigned subjects to different groups (Goette et al., 2006).

Tajfel and Turner (1979) summarized identity’s influence on behavioral decision-making into three processes: (1) categorization, the process of classifying people into a certain category based on certain characteristics; (2) identification, the process of associating oneself with certain groups; and (3) comparison, the process of comparing one’s own group with others to create favorable bias toward the group to which one belongs. Li and Claire (2020) reviewed a vast number of studies showing that social identity (or group identity or membership) often led to group bias in decisions involving different groups. Many early studies also observed that group identity manipulations can strengthen altruistic preference, reciprocity preference, and the desire to maximize social welfare among in-group members, even if group assignment is based on random and arbitrary criteria (Chen and Chen, 2011). Some researchers have shown that individuals’ social or group identity influences decision-making in cooperation games (Goette et al., 2006), dictator and response games (Chen and Li, 2009), hold-up games (Morita, and Serv’atka, M., 2013), and trust games (Wang et al., 2017). Many studies indicate that group identity induces in-group enhancement in ways that favor the in-group at the expense of the out-group (see a review by Charness and Chen, 2020). In dictator games and two-player response games, Chen and Li (2009) indicated that social identity increases subjects’ charity concerns and that participants become less envious of in-group members. As in classical social psychology experiments (Tajfel et al., 1971), they induced group identity using participant picture preferences (Klee and Kandinsky paintings). They enhanced group attachment through a combination of an online-chat problem-solving task and an other-other allocation game (in some treatments) and examined group effects using 24 self-other sequential allocation games. They found that to enhance group identity, a problem-solving stage could increase an individual’s sense of group attachment and might moderately influence behavior, while the other-other allocation had no significant effect on group identity. According to these findings, we speculate that group identity induced by categorization and enhanced by online chat’s problem-solving stage can increase group attachment and might greatly influence participants’ bidding behavior, all of which might help alleviate or resolve the overbidding problem in lottery contests.

With the recent development of neural science, brain imaging technology has provided some unique tools for revealing human cognitive and neural mechanisms. Indeed, more and more researchers have been exploring human decision-making from the neural mechanism perspective. A recent functional near-infrared spectroscopy (fNIRS) experiment organized three-versus-three-person intergroup competitions, induced in-group bonding or no-bonding control manipulation, and then measured neural activity and within-group synchronization (Yang et al., 2020). Electroencephalography (EEG) technology provides rich information about dynamic brain processes that occur during conflict detection, monitoring, and resolution. Due to its non-invasive and high-time resolution characteristics, the EEG has received much recognition in recent years for its use in cognitive neuroscience research, and it is widely used to examine human behaviors’ neural dynamics.

We focused on two event-related potentials (ERP) components; the first, N2, is a negative component that reaches a peak within 200–350 ms after stimulus presentation. Researchers have argued that N2 is mainly involved in cognitive control and that it is associated with conflict detection (van Veen and Carter, 2002; Yeung et al., 2004; Gajewski et al., 2008) and response inhibition (Hu and Zhang, 2019). Specifically, high-conflict situations can induce more negative N2 components than low-conflict situations (Fu et al., 2018); enhanced N2 refers to a higher degree of conflict (Yu et al., 2022). The N2 component has also been found to be sensitive to differences in social category cues (Ito and Urland, 2003; Derks et al., 2014). Derks et al. (2014) demonstrated that EEG responses to ethnic categories differed when participants were exposed to different social identity threats. Specifically, Muslim participants displayed greater N2 activity in response to non-Muslim (outgroup) faces than to Muslim (ingroup) faces. In addition, some evidence shows that in the N2 component, self-relevant information, such as one’s own face, country, group, or handwriting, induces smaller N2 amplitudes compared with self-irrelevant information (Chen et al., 2008; Zhou et al., 2010). In particular, self-relevant information, due to its adaptive values important to individuals, is processed preferentially and is easier to retrieve, with less consumption of top-down cognitive resources than self-irrelevant stimuli (Chen et al., 2008). The N2 component appears after stimulus onset and before manual response, suggesting that it reflects conflict processing occurring between stimulus detection and response execution.

The second ERP component, P3, is a positive component that peaks from 200–600 ms after feedback (Wu et al., 2011). P3 has usually been combined with attention and motivation; attention-capturing or motivational stimuli elicit a larger P3 component (see a review by Nieuwenhuis et al., 2005). Researchers believe that P3 may reflect the process of attention resource allocation, is relevant to social information processing in outcome evaluation, and has the largest composition in the linear part of the brain (Wang et al., 2015). Similarly to the N2 component, several studies show that P3 amplitude is sensitive to conflict adaptation effects and is enhanced by response conflict (Clayson and Larson, 2011; Groom and Cragg, 2015). Previous studies have shown that P3 amplitude is enhanced in high-conflict conditions (Gajewski et al., 2008; Schermerhorn et al., 2015). In addition, P3 has been related to evaluative categorization processes and is an index of attention to self-relevant stimuli (Gray et al., 2004). During categorization of in-group and out-group faces, previous research showed that other-race faces induced larger P2 and P3 components at the parietal region (Dickter and Bartholow, 2007). Furthermore, research in the social cognitive paradigms has demonstrated that the P3 component is sensitive to self-versus-other related processes, with self-referential information eliciting larger P3 compared with other-referential information (Knyazev, 2013).

Myriad existing EEG studies concentrate on event-related potentials (ERPs) of time-locked and phase-locked, while event-related oscillations (EROs) of time-locked but not phase-locked may provide complementary information lost in ERP results. In particular, non-phase-locked theta power modulation is exactly associated with conflict processing and is a great predictor of task condition and reaction time (Nigbur et al., 2012; Cohen and Donner, 2013). Previous studies have shown that theta band activity involves reflecting the allocation of attentional resources mediated by self-relevance (Mu and Han, 2013). Indications are that the right TPJ in the theta frequency band plays an important role in social cognition implementing processes like self–other distinction (Schiller et al., 2019). Cristofori et al. (2012) found that the amplitude of theta oscillations in the ACC region increased when social exclusion occurred. Wang et al. (2017) conducted a trust game and found that theta power was larger when interacting with outgroup proposers than with ingroup proposers. Furthermore, we pay more attention to theta power than to alpha or beta power because many studies on response conflict and self-relevant information focus on the theta frequency, in theory, the most relevant component (Nigbur et al., 2012; Cohen and Donner, 2013). In addition, theta band activity relates to cognitive control or cognitive effort. We focused mainly on the theta-band event-related spectral perturbation (ERSP) in the decision-making stage. Research has indicated that theta-frequency oscillations around the human anterior cingulate cortex (ACC) and frontal cortex—that is, frontal midline theta oscillations—may be associated with “executive attention” including self-control, internal timing, and assessment of reward in the brain (Tsujimoto et al., 2006).

Although several studies have investigated modulation of bidding behavior on performance of lottery contests or auction games (Delgado et al., 2008; Zeng et al., 2013), to the best of our knowledge, few have yet been conducted on neural responses underlying group identity’s effect on bidding behavior in lottery contests. Here, we attempted to advance this issue by analyzing modulatory effects of experimentally created and enhanced group identity on subjects’ brain activity during a lottery contest game (Tullock, 1980). Furthermore, we evaluated event-related potentials (ERP) and event-related oscillations (ERO) in the electroencephalogram. Building on previous studies’ findings, we assigned participants to almost “minimal groups” based on their preferences for Paul Klee and Wassily Kandinsky paintings (Chen and Li, 2009). Then, we compared participants’ electrocortical responses when they bid with in-group or out-group opponents. Moreover, we conducted an extended experiment to probe whether enhancing group identity affected conflict alleviation.

Thus, the present study’s main goal was to analyze ERPs (N2 and P3) and time-frequency data (theta power), the better to compare different neural responses of group identity on bidding behavior under different conditions. According to related literature (Chen and Li, 2009; Clayson and Larson, 2011; Cohen and Donner, 2013; Groom and Cragg, 2015; Fu et al., 2018), we hypothesized the following:

1. Behavioral: a participant is more likely to invest or bid lower with in-group than with out-group opponents; in Mission treatment, a participant is also more likely to bid lower after enhancement of group identity when bidding with in-group opponents.
2. ERPs: The N2 peak should be more negative when bidding with out-group than with in-group members; in Mission treatment, the N2 peak may elicit more negative amplitudes than in NoMission treatment; the P3 peak should be more positive when bidding with out-group than in-group members; in Mission treatment, the P3 peak may elicit more positive amplitudes than in NoMission treatment.
3. ERSP: Theta band power under the out-group condition is significantly greater than under the in-group condition; in Mission treatment, theta band power is significantly greater than in NoMission treatment.

## 2. Materials and methods

### 2.1. Participants

A total of 20 (12 males, 8 females; mean age: 21, 18–27 years) healthy Chinese subjects from Southwest Petroleum University of China were recruited in our EEG experiment. Sample size was determined *a priori* using G*Power 3.1 (Faul et al., 2007) in order to achieve a statistical power level of 95%, considering an alpha error of 0.05 and a small-to-medium size effect of Cohen’s d = 0.25 (Mothes et al., 2015). None of the subjects had a history of epileptic, neurological, or psychiatric disease. All participants were right-handed, with normal vision or correction. They signed written informed consent before participating in the research, which was approved by the Institutional Review Board of School of Economics and Management, Southwest Petroleum University of China. No subjects reported any adverse side effects of pain on the scalp or headaches after our experiment.

We provided subjects with a financial incentive to maximize their final payoffs. At the experiment’s conclusion, each EEG participant received a base payment of 20–40 RMB (approximately 3.1–6.1 US dollars), based on the degree of experimental coordination. Besides that, each EEG subject received another payment, an average of 40.7 RMB (approximately 6.2 US dollars) per participant, based on their own decisions.

### 2.2. Task and procedure

#### 2.2.1. Model

We implemented a simplified version of the winner-take-all lottery contest (Tullock, 1980). Two risk-neutral players are, respectively, indexed by i and by j. Each participant receives an endowment of E at the beginning of each period. The prize is worth V. In the contest, each player simultaneously chooses an investment or a “bid” from 0 to E to receive the prize. The prize is assigned to the winner, and the loser receives nothing. Let $c_{i}$ be player $i^{,}s$ investment and $c_{j}$ be player $j^{,}s$ investment. Then the probability of player i winning the prize is: $P_{i}=\frac{c_{i}}{c_{i}+c_{j}}$. If $c_{i}=0$ and $c_{j}=0$, the probability of each player winning the prize is $\frac{1}{2}$.


$$P_{i}\left(c_{i},c_{j}\right)=\{\begin{array}{r} \frac{1}{2}\;\;if\begin{array}{c} c_{i}=c_{j}=0 \end{array}\\ \begin{array}{cc} \frac{c_{i}}{c_{i}+c_{j}} & otherwise \end{array} \end{array}$$

Assuming that players are “self-interested” and risk-neutral, the expected revenue function of player i participating in the competition can be written as:


$$EU_{i}\left(c_{i},c_{j}\right)=P_{i}\left(c_{i},c_{j}\right)\left(V-c_{i}\right)+\left(1-P_{i}\left(c_{i},c_{j}\right)\right)\left(-c_{i}\right)$$

Then, we obtain the Nash equilibrium $c_{i}=c_{j}=c^{*}=\frac{V}{4}$. Substituting this optimal bid result into the expected revenue function, each player’s expected revenue is: $EU_{i}\left(c^{*}\right)=\frac{V}{4}.$

#### 2.2.2. Experimental design

**Figure 1:**
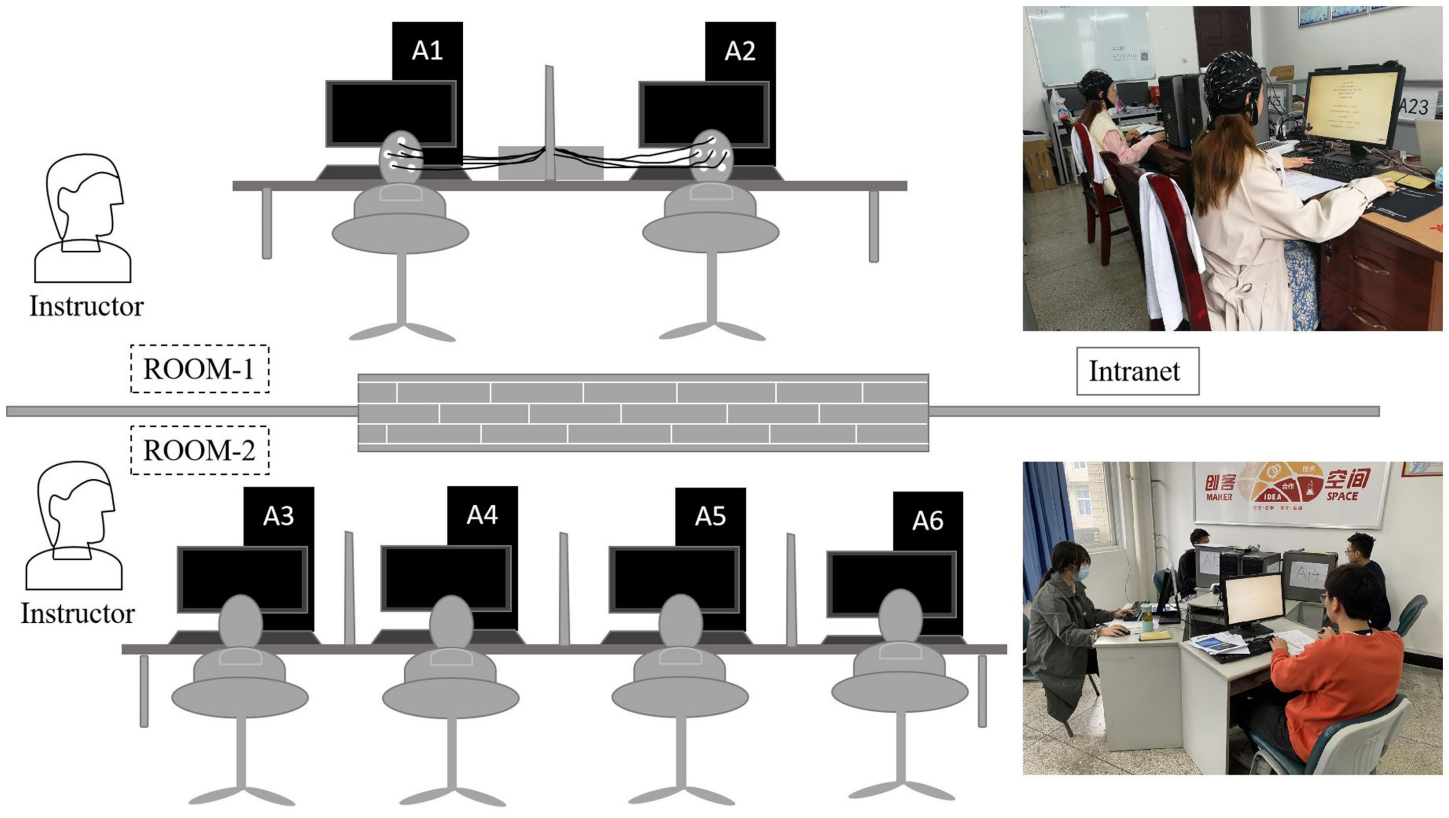
Experiments’ design sketch. EEG subjects were placed in Room 1, and interactive participants in Room 2; these two participant types were connected *via* an intranet. All participants were comfortably seated in separate areas so that they could not see other subjects’ decisions during the game. In each room, one or two instructors answered questions and monitored the process.

For this study, we adapted group identity manipulation (Chen and Li, 2009) into the lottery contest game (Tullock, 1980) to study group identity’s effect on bidding behavior. Of the two experiments conducted, one was to validate brain activity differences resulting from participants’ different bidding behaviors under in-group and out-group conditions. The other, an extended experiment, explored whether enhancing group identity had effects on conflict alleviation. We adopted the within-subjects design and employed a strategy method in which participants make contingent decisions for all nodes at which they may have to play. This method is quite useful and helpful for collecting experimental data, and we used it to elicit the subject’s bid (or investment) in different groups. Previous literature has indicated that the strategy method did not lead to different results than the standard direct-response method (Brandts and Charness, 2011). Computerized experimental sessions were run using z-Tree (Fischbacher, 2007) and E-prime (Psychology Software Tools, Sharpsburg, PA, United States) software. In the whole experiment, participants’ earnings were replaced by experimental currency units (ECU). Each period’s payoff was calculated separately, and all periods’ total payoffs were added by computer. At the end of the experiment, each subject’s total amount of ECUs was converted into RMB. Figure 1 shows the experiments’ design sketch.

As shown in Figure 1, for each session, two EEG subjects were placed in Room 1 and four interactive participants (Wang et al., 2017) in Room 2. The only differences between the four interactive subjects and the two EEG subjects were that interactive subjects’ EEG signals were not collected in a separate room nearby, and when they checked in before the experiment, the EEG subjects in Room 1 knew that real human subjects in Room 2 would interact with them during experimental tasks. These two rooms are adjacent, and the computers are connected *via* an intranet.

##### 2.2.2.1. Treatments

Our experiment included two treatments (NoMission and Mission). Each treatment consisted of two tasks: (1) a group assignment based on artwork preferences; (2) a repeated lottery contest game. However, in addition to categorization to create group identity, Mission treatment had an extra online task through an in-group chat window to enhance group identity’s effect before the repeated lottery contest game. In NoMission treatment, group identity was created only by categorization (six subjects were categorized as members of two non-overlapping groups according to picture preferences). The study adopted a within-subject design in which each subject participated in two treatments. In each treatment, each subject submitted bidding strategies for 50 periods under in-group and out-group conditions, respectively. That is, each subject input bidding strategies for 200 trials. Sessions numbered 20, including 10 for NoMission treatment and 10 for Mission treatment.

##### 2.2.2.2. Lottery task

At the beginning of the lottery contest in each period, each participant received an endowment of 125 ECU; the price of each lottery was one ECU. In the contest, players could purchase any integer amount between 0 and 125 lottery tickets. Once the ticket purchase was complete, the computer would mix the lottery tickets a participant bought with those the opponent bought into the digital basket and then randomly pick one lottery ticket whose owner won the contest (a prize of 100 ECU). The purchase cost of tickets was deducted from the initial endowment, regardless of whether the player won or lost (Su et al., 2021).

#### 2.2.3. Procedures

**Figure 2:**
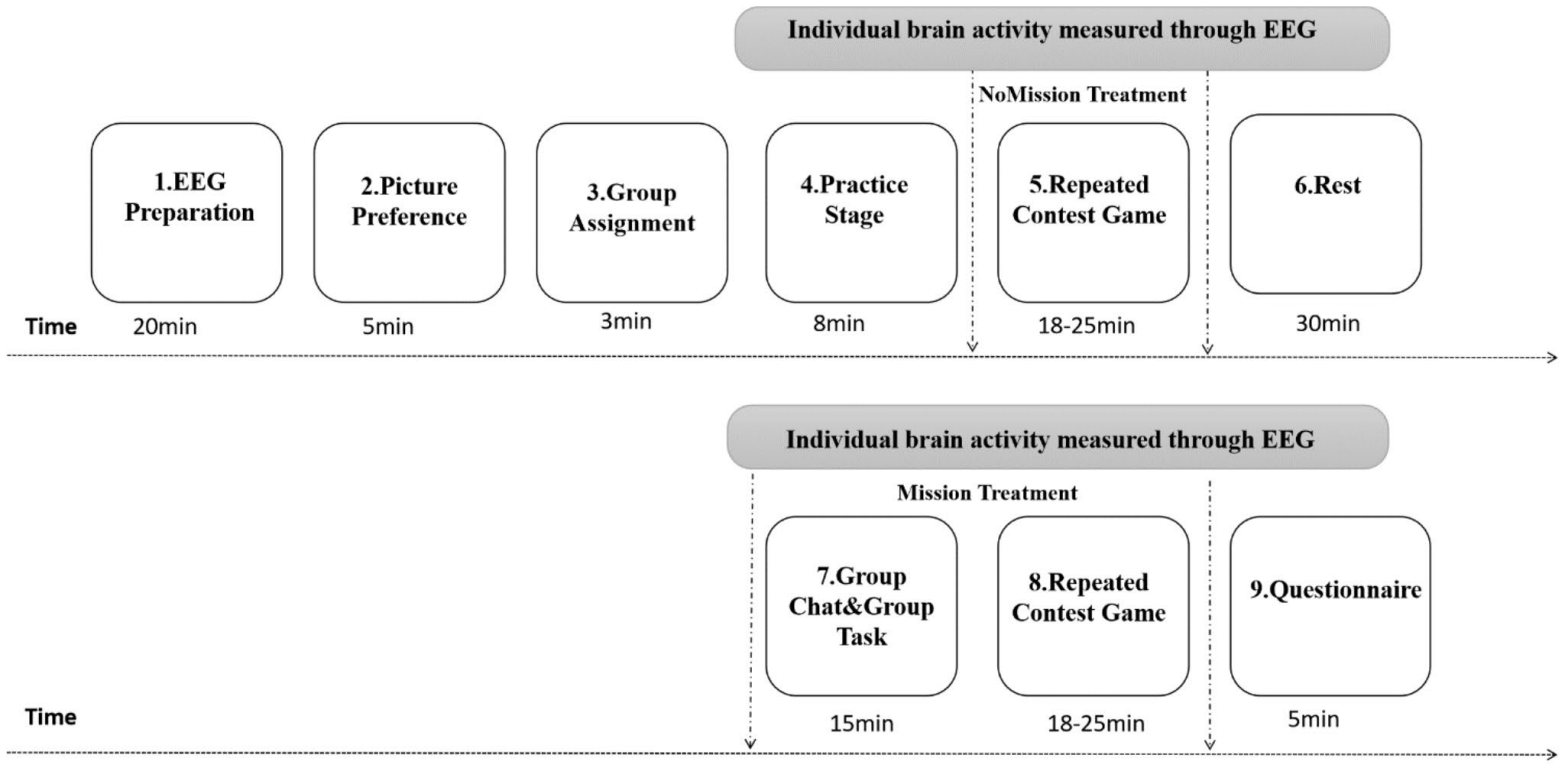
Experimental procedures.

Figure 2 presents whole stages. As shown, When EEG subjects arrived at the lab, their preparation began (e.g., hair washing, facial scrubs). Then, subjects sat comfortably on a laboratory chair, about 0.7 m away from a computer screen. They wore EEG electrode caps and completed other preparatory work.

In the second and third stages, group identity manipulation (Chen and Li, 2009) was adapted and performed through a modified version of Tajfel and Turner’s (1979) “minimal group paradigm.” Participants observed five pairs of paintings by two modern artists, Paul Klee and Wassily Kandinsky (each pair consisting of one painting by Klee and one by Kandinsky). Without revealing the artist, participants independently wrote down their favorite from each pair. According to their painting preference, subjects were assigned to one of two groups—group Klee or group Kandinsky. Following that, group membership was maintained during the entire experiment, and participants remained unaware of other group members’ identities. In short, the second stage aimed to create or induce group identity, and the third stage assigned the group.

The fourth stage was practice. All participants received written instructions and listened to an instruction broadcast prepared in advance. Furthermore, the lab instructor briefly (4–5 min) explained the experiment so that subjects would understand the experimental rules. Subsequently, the practice stage began. To minimize confusion, participants were asked to answer four questions before the experiment to test whether they completely understood the lottery game’s rules. The eight periods of contest could be implemented only after their answers were completely correct. For this practice stage, the revenue would not count in the total.

**Figure 3:**
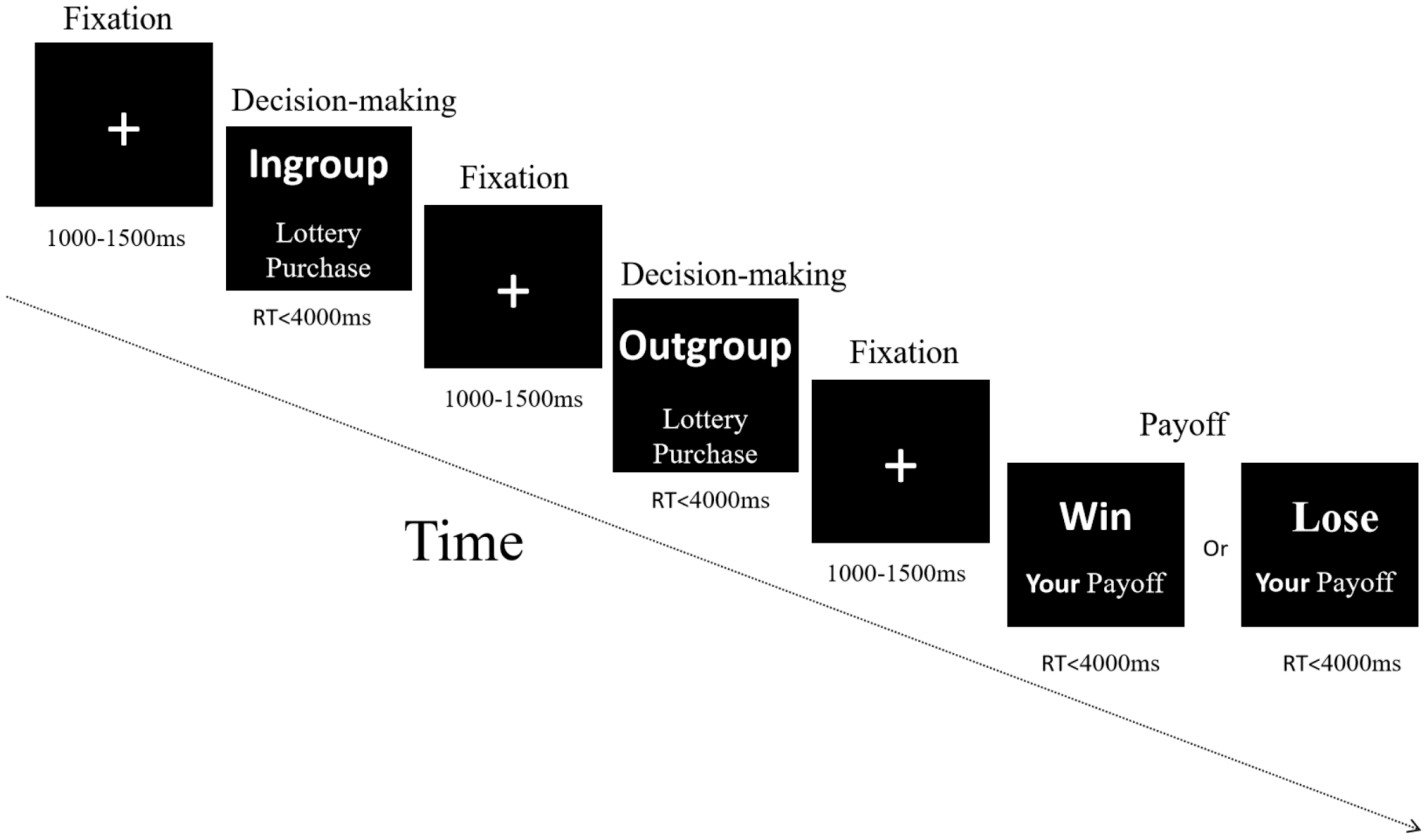
Procedures of stimulus presentation in a single period lottery contest. In each period, a white fixation cross (“+”) appeared in the screen’s center for 1,000–1,500 ms (interval randomly varied), followed by limited response times (RT) of 4,000 ms to each decision-making screen and each payoff screen. Words displayed onscreen were in Chinese.

In the fifth stage, NoMission treatment, a repeated contest game was conducted with six real persons in two rooms via intranet. Each participant submitted bidding strategies in both in-group and out-group conditions, with each condition including 50 periods, while their brain potentials were recorded using EEG. Procedures of stimulus presentation in a single period lottery contest are shown in Figure 3. In each period, a white fixation cross (“+”) first appeared at the black screen’s center for 1,000–1,500 ms (interval randomly varied). Next, participants were required to input within 4,000 ms their bid of any integer amount between 0 and 125 lottery tickets through the keyboard, under the condition that their opponents were from the in-group. When the time was up, a cross appeared again at the screen’s center for 1,000–1,500 ms. Then all players simultaneously entered the next decision screen. Similar to the former decision-making, all players input their bids through the keyboard under the condition that their opponents were from the out-group. Finally, the payoff screen appeared within 4,000 ms to inform participants of four facts: (1) whether the person paired with them was an in-group or out-group member; (2) whether the individual won or lost in the period of lottery competition: (3) how much the individual and their opponent invested; and (4) their own payoff. Opponents were randomly and anonymously matched by computer during each period, either from their own group or from the other group. Decisions had to be made in both cases, and subjects did not know their opponent’s identity, and vice versa. Experimenters tracked all decisions and payoffs using subjects’ computer ID numbers.

The seventh and eighth stages were Mission treatment, which had an extra chat task through an in-group chat window to enhance the effect of group identity; one part was choosing between the Paul Klee and Wassily Kandinsky paintings (Chen and Li, 2009), and the other was answering three skill questions (Morita, and Serv’atka, M., 2013; Masella et al., 2014). Through the chat window, subjects could communicate with other group members about the two pictures and the three skill questions. Participants especially needed to choose the artist (Klee or Kandinsky) to whom they thought the painting corresponded and answer as many skill questions as possible. All participants received messages and discussed only within their group; they could not receive information shared in other groups. All subjects were asked to follow two basic rules: (1) to be polite to each other and not to use profanity and (2) not to send messages that could identify them in any way. Correct answers from each group were summarized, and each group member with the highest overall score received an additional payment, while the other group received no payment. Then, a repeated lottery contest game was again conducted. All subjects rested between NoMission and Mission treatments, and they were forbidden to chat with each other.

The final stage consisted of a questionnaire including demographic information (e.g., gender, age, educational level, religious beliefs). Last, all subjects were paid their session earnings in private.

### 2.3. EEG data recording and pre-processing

EEG signals were recorded continuously and simultaneously using two 64-channel (Electrode arrangement according to the international 10–20 system) Neuroscan portable EEG systems. The average of two mastoids was used as a re-reference. Vertical electroophthalmogram (VEOG), which was produced by blinking and vertical eye movements, was also recorded using tiny electrodes, each placed about 1 cm above and below the subject’s left eye. Impedance between all electrodes and a subject’s scalp was less than 10 KΩ.

After EEG data acquisition, pre-processing was conducted using Letswave7 (Mouraux and Iannetti, 2008)^1^ and MATLAB (MathWorks, Natick, MA, USA) scripts. EEG data went through the following steps of pre-processing. Offline EEG time series were filtered at 0.05–35 Hz. Independent component analysis (ICA) was run to remove eye movements, and ICA components associated with eye blinks and movements were manually corrected. Subsequently, resulting data were segmented into a time window (from 1,000 ms prior to stimulus onset and finished by 2,000 ms post-stimulus interval) and baseline-corrected (200 ms prior to stimulation). Signals containing EEG amplitudes whose peak voltages exceeded ±80 μV were excluded. Last, each subject’s effective periods under each experimental condition were more than 47.

**Figure 4:**
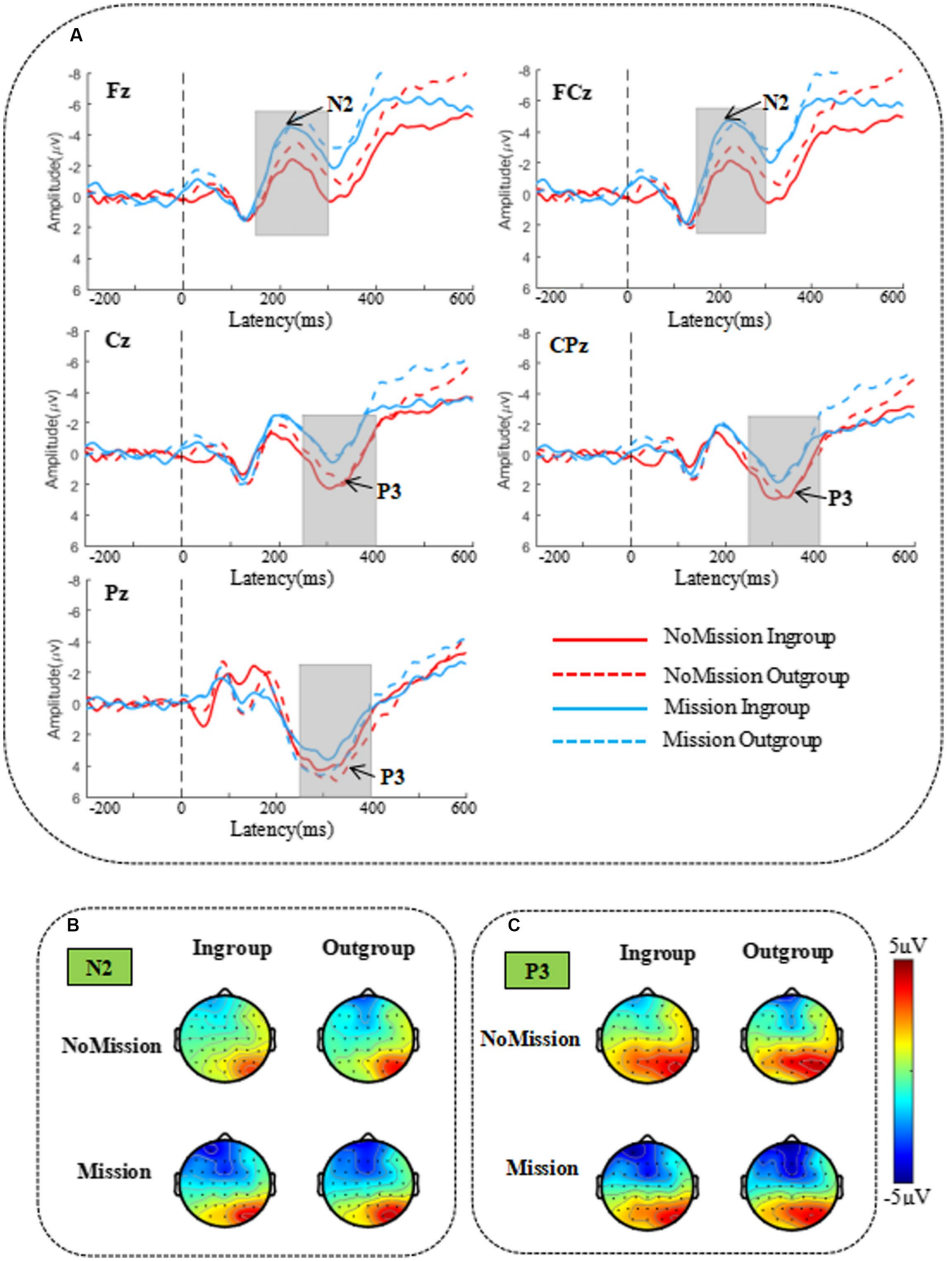
The grand-averaged waveform and topographic map of N2 and P3 components. **(A)** The grand-averaged waveform of N2 and P3 components under different conditions in five representative electrodes Fz, FCz, Cz, CPz, and Pz. The gray areas highlighted 150–300 ms, and 250–400 ms time windows were selected for analysis of peak amplitudes and latency. **(B)** Topographic maps of the N2 component under different conditions in the 150–300 ms time window. **(C)** Topographic maps of the P3 component under different conditions in the 250–400 ms time window.

Based on previous studies (van Veen and Carter, 2002; Gajewski et al., 2008; Clayson and Larson, 2011; Groom and Cragg, 2015) and visual inspection of waveforms (see Figure 4A), we selected the N2 component to measure peak amplitude and latency within 150-300 ms after stimulus onset and before the decision-making stage’s manual response. We also selected the P3 component to measure peak amplitude and latency within 250–400 ms after stimulus onset. Because myriad studies have shown that N2 is activated in the frontal, central, and even parietal sites (Deldin et al., 2000; Everhart et al., 2001; Wang et al., 2015) and that P3 is more widely distributed in the central-parietal region (Dickter and Bartholow, 2007), so we performed statistical analyses of frontal-central-parietal electrode sites. Fifteen representative electrodes in the frontal-central-parietal area were selected for statistical analysis: F1, Fz, F2, FC1, FCz, FC2, C1, Cz, C2, CP1, CPz, CP2, P1, Pz, and P2, similar to past research (Jin et al., 2017; Jing et al., 2019).

### 2.4. Time-frequency analysis

Time-frequency analysis was performed based on complex Morlet wavelet (CWT) convolution. In our study, the mother wavelet short was set to cmor 1–1.5, and time-frequency representations were explored between 3 Hz and 35 Hz. A complex sinusoidal wavelet transform program in the Letswave7 toolbox was used for neural oscillation analysis of EEG data. This program first made a single trial analysis of EEG data, then multiple trials were averaged, and finally, oscillation power under various conditions was obtained. For each estimated frequency, ERSP was displayed as an increase or decrease of oscillatory power relative to the baseline interval (−400 ms to −200 ms) according to the following formula: ER_t,f_% = [A_t,f_ − R_f_]/R_f_, where A_t,f_ was the signal power at a given time (t) and frequency (f), and R_f_ was the averaged signal power of frequency f within the baseline interval (Pfurtscheller and Lopes da Silva, 1999). Finally, the raw EEG was down-sampled to 500 Hz. The statistical difference map of ERSP data was exported in the Letswave7 toolbox, and the frequency band and time window with a significant difference were found by visual observation. Then the mean value of data in the corresponding range was exported for statistical analysis.

**Figure 5:**
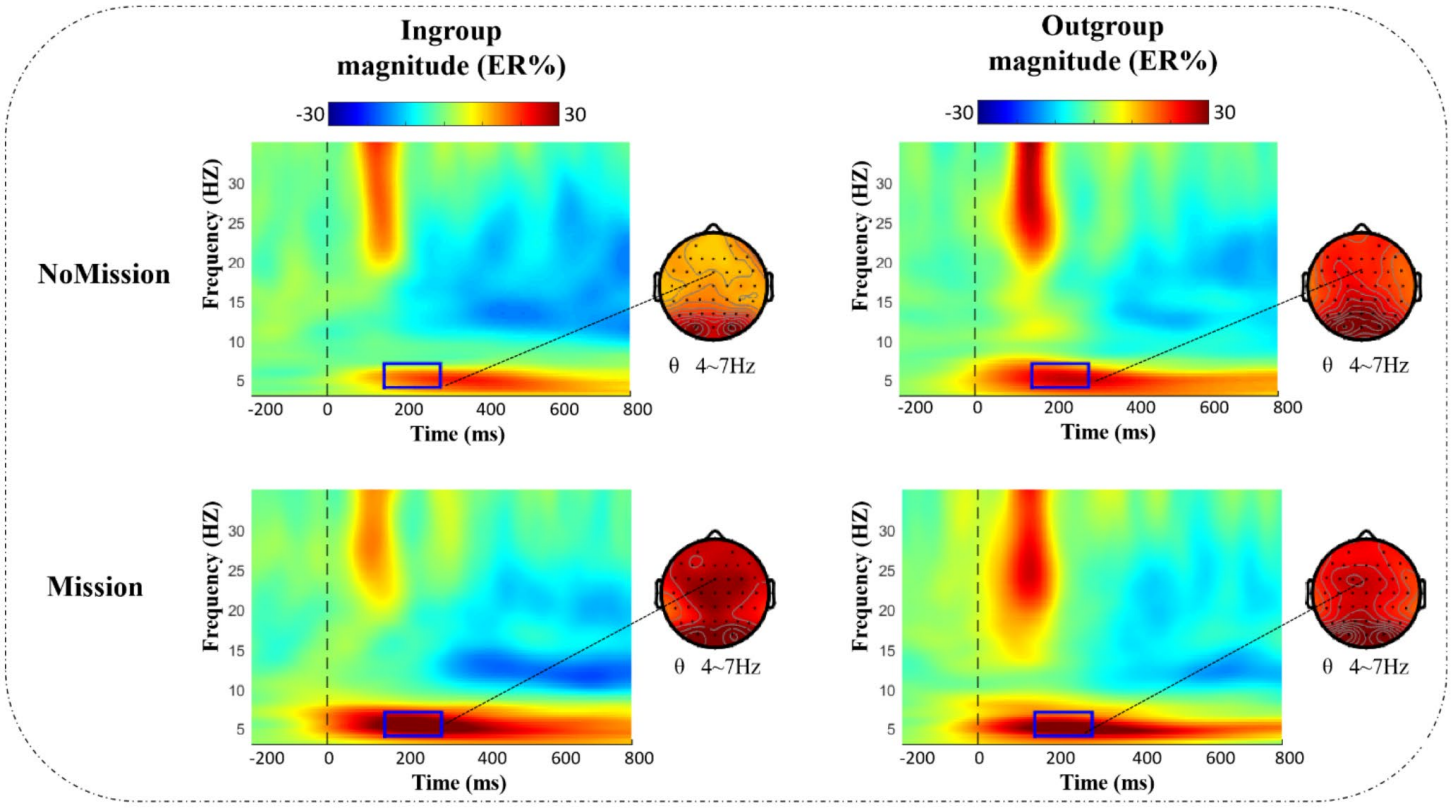
ERSP results of θ band (4–7 Hz, 150–300 ms) under different conditions at electrode FCz. Bold, dark rectangles mark the time-frequency window selected in the analysis, and dotted lines point to the corresponding topographies.

In our study, electrode FCz of the theta band (4–7 Hz, 150–300 ms) was selected as the spectral map for ERSP data (see Figure 5), and all brain data within the different frequency band/time was extracted as the topographic map. Given that topographic distribution of theta power presented in frontal-central and central-parietal sites (Sauseng et al., 2005), fifteen representative electrodes in the frontal-central-parietal area were also selected for statistical analysis: F1, Fz, F2, FC1, FCz, FC2, C1, Cz, C2, CP1, CPz, CP2, P1, Pz, and P2.

### 2.5. Data and statistical analysis

#### 2.5.1. Behavioral analysis

According to the contest model, $c^{*}$ = 25 ECU, meaning that the individual Nash equilibrium expenditure is 25 ECU. To measure the percentage of overbidding, a dummy variable was set equal to one if a subject’s bid was greater than 25 ECU and to zero if a subject’s bid was lower than or equal to 25 ECU. For behavioral data, the individual expenditure (bid or investment) and overbidding rate were analyzed using paired sample statistics. The overbidding rate was defined as the percentage of overbidding obtained by dividing the number of times the dummy variable “overbidding” equaled one by the number of periods. We calculated the individual expenditure and overbidding rate under two group conditions in two treatments, respectively. First, a Shapiro–Wilk test was conducted to examine the normality of the paired difference. Then we found that none of the paired difference data of bid and overbidding rate followed normal distribution, thus a paired Wilcoxon signed-rank test was performed.

#### 2.5.2. EEG analysis

For ERP data, we first did the Shapiro–Wilk test of N2 and P3 components in the peak and latency, and the results indicated that N2 and P3 data belonged to the normal distribution, thus a repeated measures ANOVA was used. That was, N2 and P3 were subjected to 2 (group: In-group/Out-group) × 15 (electrode: Fz/FCz/Cz/CPz/Pz/F1/FC1/C1/CP1/P1/F2/FC2/C2/CP2/P2) × 2 (identity enhancement: NoMission/Mission) repeated measures ANOVA. The Greenhouse–Geisser method was used to correct value of ps of all main effects and interactions, and the Bonferroni method was used for post-hoc comparison.

For ERSP data, a Shapiro–Wilk test of theta frequency power was conducted, and results showed that the majority of the data conformed to normality, though a few did not, with value of ps that were not particularly low. This indicated that the assumption of normality was not seriously violated, and thus the analysis of variance could be continued. Theta power was subjected to 2 (group: In-group/Out-group) × 15 (electrode: Fz/FCz/Cz/CPz/Pz/F1/FC1/C1/CP1/P1/F2/FC2/C2/CP2/P2) × 2 (identity enhancement: NoMission/Mission) repeated measures ANOVA. The Greenhouse–Geisser method was used to correct value of ps of all main effects and interactions, and the Bonferroni method was used for post-hoc comparison.

## 3. Results

### 3.1. Behavioral results

We calculated the individual expenditure (bid or investment) and overbidding rate under two group conditions in two treatments and then, respectively, performed a paired Wilcoxon signed-rank test (Table 1).

**Table 1:** Descriptive statistics and paired Wilcoxon signed-rank test results.

		In-group	Out-group	H0: In-group = Out-group
NoMission	Individual expenditure	36.187 ECU	62.176 ECU	Value of *p* = 0.0007*** (3.379)
	Overbidding rate	57.3%	83.8%	Value of *p* = 0.0007*** (3.374)
Mission	Individual expenditure	31.519 ECU	53.572 ECU	Value of *p* = 0.0012** (3.248)
	Overbidding rate	53.6%	75.8%	Value of *p* = 0.0024** (3.032)
H0: NoMission = Mission	Individual expenditure	Value of *p* = 0.0187* (2.352)	Value of *p* = 0.0859 (1.717)	
	Overbidding rate	Value of *p* = 0.1340 (1.499)	Value of *p* = 0.1545 (1.424)	

The value of *p* of each test statistic is reported in the bottom portion when we test differences between two treatments, and also in the rightmost column when we test differences between different group conditions. ***p < 0.001, **p < 0.01, *p < 0.05.

In the validation experiment, we found individual expenditure significantly lower when bidding with in-group opponents (mean ± SD, 36.187 ± 21.499 ECU) than with out-group opponents (mean ± SD, 62.176 ± 26.965 ECU) in NoMission treatment, *z* = 3.379, *p* < 0.001. Similarly, the individual bid under the in-group condition (mean ± SD, 31.519 ± 17.929 ECU) was significantly lower than under the out-group condition (mean ± SD, 53.572 ± 25.981 ECU) in Mission treatment, *z* = 3.248, *p* = 0.001. The extended experiment’s results indicated that individual expenditure was significantly higher in NoMission treatment (mean ± SD, 36.187 ± 21.499 ECU) than in Mission treatment (mean ± SD, 31.519 ± 17.929 ECU) under the in-group condition, *z* = 2.352, *p* = 0.019, while no significance was found under the out-group condition. Paired Wilcoxon signed-rank test results on the overbidding rate showed that the out-group condition (mean ± SD, 83.8 ± 21.7%) was significantly higher than the in-group (mean ± SD, 57.3 ± 36.6%) in NoMission treatment, z = 3.374, *p* < 0.001; similarly, the out-group condition (mean ± SD, 75.8 ± 28.2%) was significantly higher than the in-group condition (mean ± SD, 53.6 ± 35.4%) in Mission treatment, *z* = 3.032, *p* = 0.002. No significance was found between NoMission and Mission treatments.

### 3.2. ERP results

Figure 4 presents the grand-averaged waveform and topographic map of N2 and P3 components. The minimum peak amplitude and latency of N2 (150–300 ms) were analyzed by a repeated measurement method (ANOVA) of a three way 2 (group) × 2 (identity enhancement) × 15 (electrode). A repeated measurement method (ANOVA) of a three way 2 (group) × 2 (identity enhancement) × 15 (electrode) was applied for the maximum peak value and latency of P3 (250–400 ms). The mean and standard error (SE) for N2 and P3 components of peak and latency are displayed in Table 2.

**Table 2:** Event-related potentials components (Mean ± SE) for in-group and out-group conditions in two treatments.

	In - group	Out - group	F_Group_ (*p*)	F_Identity Enhancement_ (*p*)	F_Group × Identity Enhancement_ (*p*)
N2 Peak (μV)					
Total	−3.612 ± 0.741	−4.150 ± 0.897	0.933 (0.346)	5.407 (0.031*)	0.000 (0.990)
NoMission	−3.016 ± 0.796	−3.551 ± 0.964	0.717 (0.408)		
Mission	−4.208 ± 0.803	−4.749 ± 0.911	0.842 (0.370)		
N2 Latency (ms)					
Total	207 ± 6	213 ± 5	5.537 (0.030*)	2.695 (0.117)	4.231 (0.054)
NoMission	199 ± 6	212 ± 4	6.987 (0.016*)		
Mission	214 ± 7	214 ± 6	0.043 (0.838)		
P3 Peak (μV)					
Total	2.854 ± 0.596	2.561 ± 0.759	0.209 (0.653)	9.994 (0.005**)	0.145 (0.707)
NoMission	3.536 ± 0.573	3.414 ± 0.944	0.019 (0.891)		
Mission	2.171 ± 0.801	1.708 ± 0.660	0.477 (0.498)		
P3 Latency (ms)					
Total	313 ± 5	313 ± 6	0.004 (0.952)	0.170 (0.684)	0.175 (0.681)
NoMission	313 ± 6	316 ± 9	0.171 (0.684)		
Mission	312 ± 6	312 ± 7	0.052 (0.823)		

**p < 0.01, *p < 0.05.

#### 3.2.1. N2 component

With regard to the N2 component’s minimum peak value, statistical results showed the main effect of electrode as significant, *F* (14, 266) = 11.589, *p* < 0.001, $\eta_{p}^{2}$ = 0.379, showing that the peak of N2 increased from front to back, with the lowest peak in the frontal region (F1, Fz, and F2) and the highest in the posterior parietal region (P1, Pz, and P2). The main effect of the identity enhancement was significant, *F* (1, 19) = 5.407, *p* = 0.031, $\eta_{p}^{2}$ = 0.222, Post-hoc analysis revealed that N2 peak in Mission treatment (mean ± SE, −4.479 ± 0.806 μV) was significantly smaller than NoMission treatment (mean ± SE, −3.284 ± 0.826 μV). There was a significant interaction of group × identity enhancement × electrode, *F* (2.193, 41.675) = 5.417, *p* = 0.007, $\eta_{p}^{2}$ = 0.222. When directly comparing in-group and out-group conditions in two different treatments over five electrodes, we observed significance in NoMission treatment over electrode Fz, with the N2 peak being more negative when bidding with out-group members (mean ± SE, −4.853 ± 1.201 μV) than with in-group members (mean ± SE, −3.433 ± 0.893 μV). And we observed significance in Mission treatment over electrode P1, with the N2 peak being more negative when bidding with out-group members (mean ± SE, −3.481 ± 0.879 μV) than with in-group members (mean ± SE, −2.093 ± 0.862 μV). In addition, the simple effect test indicated that the N2 peak differed significantly between NoMission and Mission treatment under the in-group condition over the frontal-central electrodes F1, Fz, F2, FC1, FCz, FC2, C1, Cz, and C2. For example, post-hoc analysis indicated that the N2 peak in Mission treatment (mean ± SE, Fz: −6.425 ± 1.029 μV; FCz: −6.764 ± 1.059 μV; Cz: −4.395 ± 0.900 μV) elicited more negative amplitudes than in NoMission treatment (mean ± SE, Fz: −3.433 ± 0.893 μV; FCz: −3.417 ± 0.906 μV; Cz: −2.590 ± 0.921 μV) under the in-group condition over electrodes Fz, FCz and Cz, respectively. The simple effect test indicated that the N2 peak differed significantly between NoMission and Mission treatment under the out-group condition over the frontal-central electrodes F1, Fz, F2, FC1, FCz, FC2, C1, Cz, and C2. For example, post-hoc analysis indicated that the N2 peak in Mission treatment (mean ± SE, Fz: −6.898 ± 1.143 μV; FCz: −6.657 ± 1.148 μV; Cz: −4.652 ± 0.995 μV) elicited more negative amplitudes than in NoMission treatment (mean ± SE, Fz: −4.853 ± 1.201 μV; FCz: −4.407 ± 1.180 μV; Cz: −3.391 ± 1.045 μV) under the in-group condition over electrodes Fz, FCz and Cz, respectively.

With regard to latency, the main effect of electrode was significant, *F* (14, 266) = 4.080, *p* = 0.047, $\eta_{p}^{2}$ = 0.905. This also showed that N2 latency decreased from front to back, with the longest N2 latency in the frontal region (F1, Fz, and F2) and the shortest in the posterior parietal region (P1, Pz, and P2). The main effect of group was significant, *F* (1, 19) = 5.537, *p* = 0.030, $\eta_{p}^{2}$ = 0.226. Post-hoc analysis revealed that N2 latency under out-group condition (mean ± SE, 213 ± 5 ms) was significantly longer than under the in-group condition (mean ± SE, 207 ± 6 ms). A marginal significant interaction between group and identity enhancement was found, *F* (1, 19) = 4.231, *p* = 0.054, $\eta_{p}^{2}$ = 0.182. A further simple effect test indicated that N2 latency between in-group and out-group conditions in NoMission treatment was significant, *F* (1, 19) = 6.987, *p* = 0.016, $\eta_{p}^{2}$ = 0.269. Post-hoc analysis revealed that N2 latency under the out-group condition (mean ± SE, 212 ± 4 ms) was significantly longer than under the in-group condition (mean ± SE, 199 ± 6 ms) in NoMission treatment. In contrast, there was no significant difference in N2 peaks between in-group and out-group conditions in Mission treatment, *p* > 0.100.

#### 3.2.2. P3 component

As for the maximum peak value, results showed that the main effect of electrode was significant, *F* (14, 266) =5.221, *p* = 0.026, $\eta_{p}^{2}$ = 0.924, suggesting that P3 peak value increased from front to back, with the lowest peak in the frontal region (F1, Fz, and F2) and the highest in the posterior parietal region (P1, Pz, and P2). The main effect of identity enhancement was significant, *F* (1, 19) = 9.994, *p* = 0.005, $\eta_{p}^{2}$ = 0.345. Post-hoc analysis revealed that P3 peak in Mission treatment (mean ± SE, 1.940 ± 0.653 μV) was significantly smaller than NoMission treatment (mean ± SE, 3.475 ± 0.647 μV). The P3 peak was more positive when bidding with in-group members (mean ± SE, 2.854 ± 0.596 μV) than with out-group members (mean ± SE, 2.561 ± 0.759 μV), but this difference was not significant, *F* (1, 19) =0.209, *p* = 0.653, $\eta_{p}^{2}$ = 0.011.

As for P3 latency value, the main effect of electrode was significant, *F* (2.397, 45.543) = 4.406, *p* = 0.013, $\eta_{p}{\;}^{2}$ = 0.188. This specifically showed that P3 latency decreased from front to back, with the longest in the frontal region (F1, Fz, and F2) and the shortest in the posterior parietal region (P1, Pz, and P2). No other (marginally) significant interactions were found.

### 3.3. Time-frequency results

Figure 5 presents the time-frequency (TF) analysis results of theta band. Theta power (4–7 Hz, 150–300 ms) was analyzed by a repeated measurement method (ANOVA) of a three way 2 (group) × 2 (identity enhancement) × 15 (electrode). The mean and standard error (SE) for theta band power are displayed in Table 3.

**Table 3:** Theta power (Mean ± SE) for in-group and out-group conditions in two treatments.

Theta (ER%)	In-group	Out-group	F_Group_ (*p*)	F_Identity Enhancement_ (*p*)	F_Group × Identity Enhancement_ (*p*)
Total	0.210 ± 0.031	0.249 ± 0.033	2.953 (0.102)	8.166 (0.010*)	6.760 (0.018*)
NoMission	0.137 ± 0.026	0.242 ± 0.036	13.520 (0.002**)		
Mission	0.283 ± 0.041	0.257 ± 0.043	0.474 (0.500)		

**p < 0.01, *p < 0.05.

Statistical results indicated that the main effect of electrode was significant, *F* (2.690, 51.107) =4.459, *p* = 0.009, $\eta_{p}{\;}^{2}$ = 0.190, increasing from front to back, with the lowest theta power in the frontal region (F1, Fz, and F2) and the highest in the posterior parietal region (P1, Pz, and P2). The main effect of identity enhancement was significant, *F* (1, 19) =8.166, *p* = 0.010, $\eta_{p}{\;}^{2}$ = 0.301. Post-hoc analysis revealed that the theta power in Mission treatment (mean ± SE, 0.270 ± 0.037 ER%) was significantly larger than NoMission treatment (mean ± SE, 0.189 ± 0.028 ER%).

There was a significant interaction between group and identity enhancement, *F* (1, 19) = 6.760, *p* = 0.018, $\eta_{p}^{2}$ = 0.262. Direct comparison of in-group and out-group conditions in two different treatments showed a significant difference between in-group and out-group conditions in NoMission treatment, *F* (1, 19) = 13.520, *p* = 0.002, $\eta_{p}{\;}^{2}$ = 0.416. Post-hoc analysis revealed that theta power under the out-group condition (mean ± SE, 0.242 ± 0.036 ER%) was significantly larger than under the in-group condition (mean ± SE, 0.137 ± 0.026 ER%) in NoMission treatment. However, we observed no significant theta power modulation in Mission treatment, *p* = 0.500. A further simple effect test indicated that theta power between NoMission treatment and Mission treatment under the in-group condition was significant, *F* (1, 19) = 25.936, *p* < 0.001, $\eta_{p}{\;}^{2}$ = 0.577. Post-hoc analysis revealed that theta power in Mission treatment (mean ± SE, 0.283 ± 0.041 ER%) was significantly larger than in NoMission treatment (mean ± SE, 0.137 ± 0.026 ER%) under the in-group condition. However, we observed no significant theta power modulation under the out-group condition, *p* = 0.738.

## 4. Discussion

To date, considerable research from an extensive range of disciplines has investigated decision-making behavior in contests, especially its social, economic, and psychological foundations. The resulting information has great practical implications for understanding human bidding strategy in contests. Indeed, the combination of neuroscience and decision-making behavior provides an interesting perspective. Our research goals were to validate brain activity differences that resulted from participants’ different bidding behaviors under in-group and out-group conditions and to probe whether enhancing group identity had effects on conflict alleviation.

In our study, group identity was manipulated in the lab, following Chen and Li (2009), and high temporal resolution ERP technology was applied with repeated lottery contest game tasks. As one of a few brain studies to examine group identity, we provided some novel and intriguing findings. In our validation experiment, in particular, we revealed ERP and ERO correlates of competitors’ decision-making when bidding with in-group members and out-group members. N2, P3, and theta band power were used as electrophysiological indicators to explore whether group identity could induce different neural responses under different group conditions. Not surprisingly, and consistent with our hypothesis, the out-group condition exhibited more negative N2 and increased theta power. In our extended experiment, we used N2, P3, and theta band power to measure whether enhancing group identity could generate different neural responses in different treatments and tried to explain conflict alleviation. Results showed more negative N2 amplitudes, smaller P3 amplitudes, and larger theta power after enhancing group identity. Hence, our results extended existing research by finding that our method of enhancing group identity greatly influenced in-group conflict alleviation.

To the best of our knowledge, our research is the first to investigate group identity’s effect on bidding behavior in lottery contests by using the EEG technique. The study’s behavioral data showed that individual expenditure was significantly lower when bidding with in-group opponents than with out-group opponents. In line with previous studies, this result supports social identity theory (Tajfel and Turner, 1979), suggesting people’s greater tolerance of in-group members. On the other hand, our extended experiment revealed that individual expenditure was significantly higher in NoMission treatment than in Mission treatment under the in-group condition. However, no significance was found under the out-group condition. Previous studies indicated that factors identified as effective in creating salient group identity in strategic settings generally belonged to two broad categories: joint experiences and common fate. Some designs widely used to create joint experiences include communication (Cason et al., 2012), face-to-face interaction, and intergroup competition (Li and Claire, 2020). According to lower bidding results in Mission treatment, we might infer that the Klee and Kandinsky task through in-group chat is effective in enhancing group identity when bidding with in-group members. Furthermore, we expected to replicate a previous study’s results that enhanced identity could expand the conflict outside the group. However, our results did not provide any direct evidence supporting this hypothesis. In light of this finding of no significance under the out-group condition, one possible explanation is that, consistent with a previous study, enhancing group identity may have a more powerful effect on in-group “love” than on out-group “hate” (Chakravarty et al., 2016). Another possible explanation is that subjects are familiar with the rules and pay more attention to their own interests than to conflicts in a within-subject design having a short time interval. In addition, the overbidding rate in lottery contests was significantly higher than zero, showing that overbidding, previously confirmed by a considerable number of experimental researchers, also existed in this study.

In our validation experiment, note that the N2 component, which peaked at 200–350 ms after stimulus onset, differentiated competitors’ in-group from their out-group bidding choices. In particular, in the ERP literature, the N2 component is evoked by exogenous cues of novelty or conflict (Cohen and Donner, 2013). More specifically, ample evidence has suggested that N2 is related to cognitive control including, for instance, response inhibition, reaction conflict, and error monitoring (Hu and Zhang, 2019). Probably most strikingly, we observed that N2 amplitude in bidding with out-group members induced significantly more ERP negativity than in-group members over electrode Fz and P1. Previous studies have shown that self-relevant stimuli (such as one’s own country, group, or self-relevant possessive pronoun) elicited smaller N2 amplitudes than such self-irrelevant stimuli (Chen et al., 2008; Zhou et al., 2010; Xu et al., 2017). A large body of evidence has repeatedly indicated that high conflict situations can induce more negative N2 component than low conflict situations (Fu et al., 2018). This result is also consistent with those findings, suggesting that under the out-group condition, people may consume more top-down cognitive resources and exert greater cognitive control. Studies have also found that N2 is closely related to the theta band’s neural activity (Cavanagh and Frank, 2014). This study also involved event-related frequency measures in the theta band. In our study, theta power under the out-group condition, similar to N2, was significantly larger than in the in-group condition. It appears that a strong conflict in the out-group situation leads to an increase in theta power. This result might reflect that, compared with low-conflict trials, theta power increased after stimulus onset and around the time of response for high-conflict trials (Cohen and Donner, 2013). This finding is also in agreement with a study by Cristofori et al. (2012), which concluded that the amplitude of theta oscillations in the ACC region increased when social exclusion occurred. Data from our extended experiment also demonstrated that the N2 peak in Mission treatment elicited more negative amplitudes than in NoMission treatment over frontal-central electrodes. Our results similarly indicated that in Mission treatment, theta power was significantly larger than in NoMission treatment. This may be attributed to the increased complexity of investment decisions in the Mission treatment, resulting in individuals expending more cognitive resources after the online-chat problem-solving group task. Furthermore, analysis indicated that the P3 peak in Mission treatment was significantly smaller than in NoMission treatment. The P3 component is known to reflect cognitive processes related to attention allocation, stimulus evaluation, and decision-making. The decreased P3 response suggests that participants in the Mission treatment allocated fewer attentional resources compared to NoMission treatment. Overall, we found that N2, P3, and theta were modulated by group identity. This study provided a data basis for the neural mechanisms of group identity in alleviating group conflict, and also offered a theoretical basis for understanding the dynamic decision-making process and the neural oscillation characteristics of human cognitive neuroscience.

As for research limitations, first, because our experiment adopted a within-subjects design and a relatively short interval, a learning effect might have partially impacted our results. Second, no specific neurological, psychiatric, or laboratory tests were performed on individual EEG participants. Although basic demographic characteristics were collected (e.g., gender, age, educational level, beliefs), no more accurate psychological examination was conducted for participants, so the impact on the experiment could not be ruled out. Third, because our study experimentally manipulated the lottery contest context, investigating whether real-life identity similarly modulates bidding strategy would be intriguing and necessary in future research. Finally, a within-subject design is very likely to elicit changes between treatments due to experimenter demand effects.

As outlined above, we provide novel findings that shed new light on how group identity influences individual bidding behavior by modulating brain processes of decision-making. Many studies present some mechanisms proven effective for enhancing within-group cooperation and also some conflict resolution mechanisms that can mitigate and de-escalate between-group conflicts (Sheremeta, 2018). These include punishment of free-riders, within-group communication, and feedback about relative group performance. Some conflict resolution mechanisms include between-group communication, side-payments, and coordination. However, few studies have indicated a mechanism of social or group identity effect on human competitive behavior. Thus, our findings will not only enrich theories on group identity but also provide valuable guidance for designing effective interventions to reduce in-group conflict. Some outstanding researchers have also noted that the study of social identity’s effects on an individual’s separate or group behavior is still in its very early stages (Charness and Chen, 2020; Li and Claire, 2020) and that these aspects need further examination. So far as we know, most of these previous studies have concentrated on resolving conflicts between individuals, and our study also aims to utilize the team setting to gain insight into individual behavior through lottery contests. Maybe our further research should pay close attention to group level contests.

## 5. Conclusion

In conclusion, this study applied the EEG technique to investigate group identity effect on bidding behavior in lottery contests. In line with the relevant literature, the study confirmed significant differences in bidding behavior between in-group and out-group settings, suggesting in-group love when bidding with in-group members. We further explored whether enhancing group identity had effects on conflict alleviation; findings showed that competitors had a higher degree of identification with their in-group rivals after adopting the method of enhancing group identity, whereas no significance was found under out-group conditions. This might result from the within-subject design and the individual level contest. All subjects were familiar with the rules and paid more attention to their own interests than to out-group conflicts. In the future, we will focus our research on the group level of contest and on more methods of enhancing group identity.

## Data availability statement

The raw data supporting the conclusions of this article will be made available by the authors, without undue reservation.

## Ethics statement

The studies involving human participants were reviewed and approved by this study received ethics approval from the Institutional Review Board of School of Economics and Management, Southwest Petroleum University. The patients/participants provided their written informed consent to participate in this study.

## Author contributions

SH, PJ, and ZX: methodology. SH, PJ, ZX, and WX: software, data curation, formal analysis, writing original draft, drawing figures, and writing review and editing. SH, PJ, ZX, WX, LL, QX, and LQ: experiment organization. All authors contributed to the article and approved the submitted version.

## Funding

This work was supported by Humanities and social science special fund of Southwest Petroleum University of China (2022-2023RW004), Humanities and social science projects supported by Ministry of Education of China (grant number 20YJC790120).

## Conflict of interest

The authors declare that the research was conducted in the absence of any commercial or financial relationships that could be construed as a potential conflict of interest.

## Publisher’s note

All claims expressed in this article are solely those of the authors and do not necessarily represent those of their affiliated organizations, or those of the publisher, the editors and the reviewers. Any product that may be evaluated in this article, or claim that may be made by its manufacturer, is not guaranteed or endorsed by the publisher.

## Footnotes

^1^ https://letswave.cn
